# Social Isolation and Sleep: Manifestation During COVID-19 Quarantines

**DOI:** 10.3389/fpsyg.2021.810763

**Published:** 2022-01-10

**Authors:** June J. Pilcher, Logan L. Dorsey, Samantha M. Galloway, Dylan N. Erikson

**Affiliations:** Department of Psychology, Clemson University, Clemson, SC, United States

**Keywords:** social isolation, sleep, social relationships, COVID-19, loneliness, review

## Abstract

Although researchers have investigated the impact of social isolation on well-being, the recent quarantines due to COVID-19 resulted in a social isolation environment that was unique to any examined in the past. Because sleep is one of the endogenous drives that impacts short and long-term health and well-being, it is important to consider how social isolation during the COVID-19 government-mandated quarantines affected sleep and sleep habits. A number of researchers have addressed this question during the last 2 years by examining several concepts related to possible changes in sleep during the quarantines. To best understand these recent results, the current mini review provides a background on the pre-pandemic literature on the effects of social isolation and loneliness with a focus on sleep and then summarizes the recent literature on sleep and sleep habits. In general, sleep was negatively impacted for many people during the pandemics but not all. One group that seemed to benefit from the pandemic in terms of sleep patterns, were younger people who could more easily adapt their sleep times to match their internal chronobiology. Given the potential broad impact of sleep on health and well-being, better understanding how social isolation impacts sleep is an important consideration for individuals, work organizations, and governments.

## Introduction

Social isolation can arise from many causes. Disruptions to our daily social connections (e.g., job loss) result in people adapting to some level of social isolation. Quarantine procedures used to control virus outbreaks can result in extended social isolation. For example, numerous citywide quarantines were put in place in China and Canada during the 2003 outbreak of severe acute respiratory syndrome (SARS). In the United States, individuals with SARS were placed in individual quarantine. Similarly, many villages in west African countries were quarantined in the 2014 Ebola outbreak (Brooks et al., 2020). Since the new SARS outbreak, COVID-19, in December 2019, many people in numerous countries have been required to quarantine for weeks to months where they were obligated to work at home, home-school children, and drastically decrease social interaction resulting in prolonged periods of enforced social isolation.

The impact of such widespread social isolation is not yet well understood. Some research has, however, focused on the potential impact of meaningful social relationships in humans. The association between positive social relationships and health has been clearly established. Individuals who are well integrated into society typically display lower mortality, lower rates of chronic diseases, and healthier overall behavior (House et al., 1988). Similarly, being socially connected positively impacts psychological, emotional, and physical well-being (Uchino, 2006). A meta-analytic summary of the literature found that inadequate social relationships is linked with an increase in risk of premature mortality that is similar to well-established health-risk factors such as decreased physical activity and increased obesity (Holt-Lunstad et al., 2015).

Good interpersonal relationships could help enable social influence on individual choices and behaviors by providing a structure that encourages individuals to choose better health-related behaviors (Umberson, 1987). For example, social relationships can help one maintain a more consistent sleep routine that can result in better stress management and long-term health benefits. However, the functionality of this type of social structure could be undermined by a quarantine-like scenario such as those implemented to control the spread of COVID-19.

The purpose of this mini review is to assess recent findings pertaining to sleep and sleep habits under COVID-19 quarantine conditions within the broader context of the social isolation research area. To identify the relevant literature, we used Google Scholar and PsycInfo. We also searched the citations within the literature we identified and the “cited by” index in Google Scholar to locate additional relevant studies. Together, these search methods provided a substantial base of literature to inform our assessment of social isolation and sleep.

## Social Isolation and Loneliness

Research examining the impact of social isolation is often intermixed with research on perceived loneliness. While the concepts of social isolation and loneliness are sometimes used interchangeably, they can be viewed as two separate scientific constructs. Perceived loneliness is defined by one’s inability to fulfill personal social desires (Ernst and Cacioppo, 1999; Cacioppo et al., 2010). Individuals are at greater risk of experiencing perceived loneliness when they have few social networks, conflicts in their current family or intimate relationships, or social connections that are not close enough to be considered a social relationship (Dykstra and Fokkema, 2007; van Tilburg et al., 2015; Gierveld et al., 2018). It is important to note that an individual can be lonely, but not be socially isolated and vice versa (Victor et al., 2000; Cacioppo et al., 2009; Gierveld et al., 2018). While the focus of the current paper is on social isolation, it is important to note that feelings of loneliness are likely part of the broad impact of social isolation resulting from the COVID-19 quarantines.

Although one can be socially isolated and not experience the emotion of loneliness, this is unlikely for most people (Victor et al., 2000; Gierveld et al., 2018). By nature, humans are social entities and when they are isolated from human contact or experience feelings of loneliness, health risks and sleep problems increase (Cacioppo et al., 2000, 2011; Robins et al., 2018; Griffin et al., 2020). In the case of social isolation due to the COVID-19 quarantines, it is likely that many individuals maintained some level of family connections but experienced a decrease in other social contact. In addition, many individuals could have maintained some level of social relationships using social media (Pancani et al., 2021). It seems likely that most individuals had some level of social contact during the COVID-19 quarantines, nonetheless most would have experienced a dramatic decrease in their normal social interactions.

Research suggests that socially isolated individuals are at a greater risk for increased stress and higher mortality and morbidity (House et al., 1982, 1988; Umberson, 1987; Brummett et al., 2001; Cacioppo et al., 2011). Some research equates experiencing social isolation with the same health risks as smoking 15 cigarettes or drinking six alcoholic beverages a day (House et al., 1982; Brummett et al., 2001; Holt-Lunstad et al., 2015; Robins et al., 2018). Social isolation is also highly comorbid with mental disorders like depression, anxiety, and dementia (Ernst and Cacioppo, 1999; Cacioppo et al., 2000; Robins et al., 2018). However, research also indicates that interaction with as little as one other individual can greatly reduce the health risks associated with social isolation (Cacioppo et al., 2000; Brummett et al., 2001), suggesting that the specific characteristics of the social isolation environment can affect the broader impact on individuals.

The exact social isolation conditions resulting from COVID-19 quarantines varied across different individuals and countries. Some individuals could have been isolated with their families while other individuals could have been living alone but using social media to maintain some social contact. Similarly, some countries repeated quarantines over time while other countries had longer, sustained quarantines in place. As such, the social isolation with the resultant feelings of loneliness experienced during COVID-19 quarantines is unique and presents an environment unlike the types of social isolation seen in previous studies.

## Sleep and Sleep Habits

Pre-COVID-19 pandemic studies have shown that social isolation negatively impacts sleep (Cacioppo et al., 2002; Kurina et al., 2011). Furthermore, a recent review of the pre-pandemic literature on social relationships and sleep concluded that increased quantity and quality of mutually supportive relationships are positively related to sleep (Gordon et al., 2021). It is also well established that sleep and sleep habits can be negatively impacted by psychosocial stress (Åkerstedt, 2006) as occurs during prolonged social isolation as seen in the COVID-19 quarantines (Yuksel et al., 2021).

Sleep is a health behavior that is highly predicated on a consistent routine and thus, could be compromised as a result of the changes in lifestyle during a quarantine. The implication of this relationship could be significant, given the evidence in the scientific community that links poor sleep to poor health outcomes. Sleep disturbances, unrelated to an underlying medical condition, are associated with increased cardiovascular disease, cancer, and obesity as well as to all-cause mortality (Cappuccio et al., 2010). Sleep disturbances also negatively impact psychological functioning, immune response, and mood regulation (Medic et al., 2017). In addition, sleep habits are a better predictor of mental health and well-being than other health-related behaviors such as physical activity and diet (Wickham et al., 2020).

Sleep loss can also have more acute effects on health. For example, a single night of simulated shift work results in higher blood pressure among young adults at risk for hypertension (McCubbin et al., 2010) and alters respiratory sinus arrhythmia when completing complex tasks (Walker et al., 2009). As such, maintaining good sleep habits and adequate sleep would be an important component of good mental and physical health both in the short-term, such as during a quarantine, but also lifelong.

It is important to note that sleep quality is at least as important to good health as sleep quantity. Sleep quality is often the better predictor of mental health and well-being (Wickham et al., 2020) and is more closely associated with health, affect, and life satisfaction than sleep quantity (Pilcher et al., 1997) particularly in persons sleeping between 6 and 8 h a night. In addition, both social isolation and perceived loneliness negatively impact sleep quality (Matthews et al., 2017; Cho et al., 2019). It has also been shown in older individuals that social isolation predicts poor sleep quality 6 years in the future (Yu et al., 2018). Therefore, understanding the interaction between sleep and social relationships is important in understanding how humans’ social nature impact our health and well-being (Cacioppo et al., 2011; Robins et al., 2018).

## Social Isolation and Sleep During COVID-19

Understanding the impact of government-mandated quarantines that extended across many countries and millions of people on sleep is crucial due to the many links between sleep, health, and well-being. Because modern technology has enabled forms of communication that make total isolation less likely, the COVID-19 quarantines may not have resulted in total social isolation for many people, however, the quarantines would have caused unique challenges leading to changes in daily life for most people. Furthermore, recent research indicates that people experienced increased feelings of depression and anxiety during the COVID-19 quarantine (Peterson et al., 2021) which could negatively impact sleep and sleep habits for many individuals.

### Meta-Analyses and Large Multi-Country Studies

A number of review articles and multi-country studies have focused specifically on sleep and sleep patterns during the COVID-19 quarantine. One meta-analysis examining sleep habits across 13 countries found that 35.7% of the participants experienced sleep disturbances (Jahrami et al., 2021). Another meta-analysis examined gender differences in sleep problems across 19 countries and found that 24% of female participants and 27% of male participants experienced sleep problems during the quarantines (Alimoradi et al., 2021).

Several multi-country studies included specific information about sleep habits that was not easily captured in the meta-analyses. One multi-country study found that over 50% of the participants delayed their sleep and wake times with more than a third of the participants reporting increased sleep disturbances (Yuksel et al., 2021). In addition, they concluded that poorer sleep was associated with increased depression and anxiety symptoms. Another multi-country study reported that equal numbers of participants indicated no change in sleep as worsening sleep (Mandelkorn et al., 2021). In this study, the people most likely to report increased sleep disturbances were women, people between the ages of 31 and 45, and people who were less physically active. One multi-country study found that sleep was seen as less restful and of lower quality during the quarantine than prior to the quarantine and that decreases in sleep quality were negatively correlated with perceived social isolation, depression, and anxiety (Salah et al., 2021). Finally, a multi-country study that included many high school students found that most changes in sleep patterns occurred within the first 2 weeks of the quarantine (AMHSI Research Team et al., 2020). More specifically, they reported that the differences between sleep duration on weekdays versus weekends disappeared with total sleep time increasing by about one hour during the quarantine particularly for the adolescents.

These large-scale studies provide a good foundation for understanding the impact of social isolation during the COVID-19 quarantine on sleep and sleep habits. These studies reported that people often indicate worse sleep quality or sleep problems during the quarantine. However, there were also some people, particularly older adolescents, who increased their sleep time. In addition, there were differing reports on the prevalence of sleep problems in females and males.

### Primary Studies

Other studies, though individually less encompassing than the reviews and multi-country studies, provide important information about the impact of social isolation during the COVID-19 quarantines on sleep. In this section, we focus on primary studies that were published more recently and were not captured in the meta-analyses described above to provide a broad picture of what the current literature is showing.

Many sleep scientists used sleep quality or similar constructs to document changes in sleep during the COVID-19 quarantines. Many studies across numerous countries reported a worsening of sleep quality, increasing sleep problems, or increasing problems with insomnia during quarantine conditions (Blume et al., 2020; Peretti-Watel et al., 2020; Pinto et al., 2020; Voitsidis et al., 2020; AlRasheed et al., 2021; Cellini et al., 2021; Conte et al., 2021; Hisler and Twenge, 2021; Hyun et al., 2021; Marelli et al., 2021; Martínez-de-Quel et al., 2021; Robillard et al., 2021). However, some studies found that sleep quality did not worsen across all participants (Benham, 2020; Gao and Scullin, 2020; Kocevska et al., 2020; Tahara et al., 2021). Of these studies, a common trend was that younger people or college students reported less sleep problems during the quarantine (Benham, 2020; Tahara et al., 2021).

In addition to quality-type measures, many researchers examined changes in sleep patterns during the COVID-19 quarantines. Delays in sleep timing which frequently included a concomitant increase in sleep quantity were commonly found across many countries (Benham, 2020; Blume et al., 2020; Sinha et al., 2020; Wright et al., 2020; Cellini et al., 2021; Conte et al., 2021; Robillard et al., 2021; Tahara et al., 2021). The delays in sleep timing included later bedtimes, mid-sleep times, and wake-up times often resulting in less social jetlag (where sleep times differ between workdays and non-workdays). One study found that the number of college students reporting seven or more hours of sleep a night increased from 84 to 92% for weekdays during the quarantine (Wright et al., 2020). Not surprisingly, there was variability in the responses from participants about time in bed during the pandemic. One study reported that although average wake time was delayed, some participants reported a decrease in total sleep time (Robillard et al., 2021) while a different study focusing on adults over 60 years old found that about 27% of participants reported more sleep than usual while about 15% reported less sleep than usual during the pandemic (Emerson, 2020). Another study found that average time asleep did not change during the pandemic, however, there was greater prevalence of both shorter and longer sleep than the recommended 7–8 h a night (Hisler and Twenge, 2021).

Another area of sleep-related research during the COVID-19 quarantines concerned the potential impact of variables related to the characteristics of the individual participants. This included differences between females and males, personality types, stress, and mood variables. In general, women were more likely to experience worse sleep quality and worse insomnia than men across many countries (Pinto et al., 2020; Voitsidis et al., 2020; Cellini et al., 2021; Marelli et al., 2021; Robillard et al., 2021). In addition, women were more likely to experience increased feelings of distress and loneliness during the quarantines (Losada-Baltar et al., 2021). In contrast, one study found that although women reported more anxiety than men, their sleep quality did not differ (Bigalke et al., 2020).

Lower quality sleep and insomnia were correlated with depressive symptoms and feelings of loneliness (Voitsidis et al., 2020; Hyun et al., 2021), feelings of anxiety (Xiao et al., 2020), a more negative mood (Ingram et al., 2020; Cellini et al., 2021), and negative affect and worry (Kocevska et al., 2020). Furthermore, some studies divided the participants into groups depending on sleep-related variables or personality-type variables. One study divided their participants into three groups based on sleep quantity (reduced or extended) and delayed sleep times (Robillard et al., 2021). They concluded that those participants experiencing a reduction in sleep quantity or delayed sleep times also experienced more stress, anxiety, and depression during the quarantines. Another study divided their participants into three groups based on adaptive-type personality profiles (Ahmed et al., 2021). They found that persons with highly adaptive personalities exhibited less perceived stress and better sleep quality while persons with a maladaptive personality profile experienced the highest perceived stress and poorer sleep quality.

## Conclusion and Implications

The drastic increase in levels of social isolation during the COVID-19 quarantines was accompanied by changes in sleep. Sleep quality suffered, particularly in women. Interestingly, the timing of sleep changed with some people, especially younger adults, shifting their bed and wake up time to later in the day while often increasing the time asleep. This suggests that many people curtail their sleep to meet the time demands of their workplace or education settings and that the quarantines provided flexibility, thus allowing some individuals to better match their sleep to their chronobiological drives. It is also important to note that not everyone experienced an increase in sleep quantity during the quarantines. Some people, particularly those who reported feelings of depression, anxiety, loneliness, or stress, experienced less sleep as well as worse sleep quality.

As noted earlier, social isolation could include feelings of loneliness for some people. Unfortunately, this manuscript could not address this issue since few studies on sleep during the COVID-19 quarantines examined loneliness. Those studies that did suggested that feelings of loneliness were related to increased incidences of disturbed sleep. However, future research is needed to better document the potential impact of loneliness on sleep under social isolation conditions.

As would be expected, sleep and sleep habits varied across individuals during the COVID-19 quarantines. Some of the differences in sleep disruptions were related to individual characteristics such as differences between females and males or a different adaptation in sleep behaviors for younger adults than other adults. There are other variables that could have moderated the impact of social isolation on sleep such as social support. Studies indicate that during the COVID-19 quarantines perceived social support served as a buffer for the negative impact of the quarantines on sleep, stress, anxiety, and depression (Grey et al., 2020; Li et al., 2020; Xiao et al., 2020). A lack of social support can also cause a decline in self-control ability (Pilcher and Bryant, 2016), which in turn could lead to poorer decisions regarding sleep habits (Pilcher et al., 2015). This sequence of events could contribute to worse sleep under social isolation conditions for individuals who experience less social support. Another potential moderating variable is exercise which has been shown to be related to sleep quality and quantity (Kovacevic et al., 2018; Pilcher et al., 2021). Unfortunately, exercise was not a common measure for the quarantine-based studies on sleep and, thus, could not be assessed here. Other potential moderating variables could be family and work obligations, preexisting medical conditions, or neurological conditions. Unfortunately, these types of constructs were not commonly measured in the quarantine-based studies on sleep. Additional research is needed to document the interactions between potential moderating variables and the impact of social isolation on sleep.

The literature reviewed here suggests that a complex, multi-dimensional relationship exists that connects social integration and sleep. In general, social isolation during the quarantines negatively impacted sleep for many individuals which could then have short- and long-term consequences on health. Given that interventions in one health area could positively impact other health areas (Rajkumar, 2020), it is important to consider the potential impact of developing interventions that address the range of sleep problems that can emerge during social isolation conditions. This can include interventions from governments, workplaces, and health-care professionals that incorporate educational efforts, treatment of insomnia, chronobiological therapies, and cognitive-behavioral therapies that can help individuals more readily adapt to social isolation.

The current mini review supports the importance of understanding the broader impact of social isolation on sleep as well as the need for additional research. It is important to note that social isolation could occur in a variety of settings other than a pandemic, including isolation because of personal decisions to withdraw from others, disruption in a normal social group such as when losing a job, mental disorders, retirement, aging, and when working primarily from home. Government, workplace, and health-care interventions are needed that are broadly accessible by the public to assist individuals in better managing their health behaviors including their sleep habits when experiencing social isolation.

## Author Contributions

JP completed the final drafts of the manuscript. All authors were part of the conceptualization of the manuscript and worked on earlier drafts.

## Conflict of Interest

The authors declare that the research was conducted in the absence of any commercial or financial relationships that could be construed as a potential conflict of interest.

## Publisher’s Note

All claims expressed in this article are solely those of the authors and do not necessarily represent those of their affiliated organizations, or those of the publisher, the editors and the reviewers. Any product that may be evaluated in this article, or claim that may be made by its manufacturer, is not guaranteed or endorsed by the publisher.
